# Ivermectin and Moxidectin Can Incapacitate Different Strains of the Common Bed Bug Cimex lectularius L.: A Study

**DOI:** 10.7759/cureus.6714

**Published:** 2020-01-20

**Authors:** Johnathan M Sheele, Elizabeth Lesser, Xiaolin Li, Danie Schlatzer, Gale Ridge

**Affiliations:** 1 Emergency Medicine, Mayo Clinic, Jacksonville, USA; 2 Emergency Medicine, Case Western Reserve University, Cleveland, USA; 3 Emergency Medicine, The Connecticut Agricultural Experiment Station, New Haven, USA

**Keywords:** bed bug, cimex lectularius, moxidecin, ivermectin, high performance liquid chromatography, mass spectroscopy, ectoparasite, xenointoxication, treatment

## Abstract

The common bed bug *Cimex lectularius* L. *(C. lectularius)* is a hematophagous ectoparasite that has recently resurged in many western industrialized nations, in part due to pesticide resistance. Using a laboratory feeding system, we found that the antiparasitic drugs ivermectin and moxidectin did not show higher incapacitation rates in pyrethroid-resistant strains of *C. lectularius* compared to a pyrethroid-susceptible strain. Additionally, we developed a high-performance liquid chromatography (HPLC) and mass spectroscopy (MS) assay to measure the concentrations of ivermectin inside *C. lectularius* and found that ivermectin persists in the insects for up to one month. HPLC/MS will be useful in understanding the pathophysiology behind the long-term morbidity observed in *C. lectularius* that consumes a sublethal dose of ivermectin.

## Introduction

The common bed bug *Cimex lectularius* L. (C. lectularius) is an obligate hematophagous insect that preferentially feeds on humans. *C. lectularius* infestations have increased in recent decades in many industrialized nations including the US [1]. Integrated pest management (IPM) is used to control bed bug infestations by reducing harborage areas and by using heat and/or pesticides [2]. However, *C. lectularius* resistance to commonly used pesticides, particularly the pyrethroid-class of pesticides, is contributing to the bed bug resurgence, and thus new approaches in controlling the insects are needed. A successful approach that was used to treat other human ectoparasites (e.g., *Sarcoptes scabiei var. hominis*, *Pediculosis capitis*, *Pediculosis corporis*, and *Pediculosis pubis*) involves the use of an oral antiparasitic drug such as ivermectin. Ivermectin is administered to about 250 million people annually, mostly as part of mass drug administration programs in developing countries [3]. The antiparasitic drugs ivermectin and moxidectin have limited human toxicity at therapeutic doses, and both have been shown to kill bed bugs that consume blood meals spiked with these drugs in a dose-dependent manner [4-10]. Bed bugs that survive blood meals containing ivermectin or moxidectin suffer long-term harm including reduced fecundity, inhibition of nymphal molting, delays in blood meal digestion, and reduced locomotion [5-10]. However, previous research on ivermectin has involved laboratory-adapted bed bugs with an unknown sensitivity to the pyrethroids and neonicotinoids; and there has only been one study using moxidectin and the Irvington stain, which is known to have moderate resistance to pyrethroids [6-8]. Because most *C. lectularius* in the US are now pyrethroid-resistant, an objective of this study was to explore ivermectin and moxidectin toxicity in different *C. lectularius* strains including previously reported pyrethroid-resistant and pyrethroid-susceptible insects [11].

Ivermectin is known to act at the invertebrate glutamate-gated chloride channel, causing nerve and muscle hyperpolarization [12]. The pharmacotoxicity of a sublethal dose of ivermectin against *C. lectularius* is unknown but could be multifactorial including the persistence of ivermectin in the insect, changes in gene expression, or damage to *C. lectularius* organs [6-10]. Therefore, an additional objective was to quantify ivermectin levels in fed bed bugs.

## Materials and methods

Insects

The Harlan strain of *C. lectularius* has been maintained under laboratory conditions since the 1970s and is susceptible to the pesticides used in bed bug management including the pyrethroids. The Anderson strain was obtained in 2007 from an apartment in New Haven, CT, and the Ridge strain was obtained from several infested apartments in 2009. Both strains have been maintained under laboratory conditions. Neither the Anderson nor Ridge strains have been characterized to determine their level of pyrethroid resistance, but both strains were included in the current assessment as both have been used previously to study the effects of ivermectin and moxidectin [5,8,11]. The Indy strain was collected in 2008 from Lugar Tower, Indianapolis, IN. The Irvington strain was obtained from multiple apartments during 2012-2013 in Irvington, NJ. Both the Indy and Irvington strains are recorded to have moderate levels of pyrethroid resistance [13].

Design

Two separate feeding experiments were performed in which *C. lectularius* from each strain were randomly assigned to feed on blood containing different concentrations of ivermectin, moxidectin, or a dimethyl sulfoxide (DMSO) control. Bed bugs were kept in 1.5-mL test tubes. Each test tube had a piece of paper upon which insects perched. The cap for each tube had a hole drilled into the top and a piece of sheer fabric glued to the surface so that insects could be fed. The insects were fed by putting a piece of parafilm over the cap and inverting the test tube into a petri dish containing warmed blood. The blood was kept warm on a hot plate set at 40 °C. The percentage of bed bugs that fed was not recorded and unfed insects were discarded. All insects were fed a single blood meal during their observation period.

In the first experiment, the insects were fed on 0, 2.5, and 25 ng of ivermectin or moxidectin, and the incapacitation rates were observed on days 11 and 23. In the second experiment, the insects were fed on 0, 1, 25, or 50 ng of ivermectin or moxidectin, and the incapacitation rates were recorded on days 3, 16, and 33. An assessment of bed bug fecundity was also done on day 33 of the second feeding experiment. Bed bugs were considered incapacitated if the insect was dead, paralyzed, unable to cling, or largely immobile upon tactile simulation. Insects that appeared unaffected or only slightly harmed were not considered to be incapacitated.

Materials

A 1.0% suspension of ivermectin (Noromectin w/v Multi Injection Solution for Injection; Norbrook Laboratories, Newry, UK) was diluted to a final concentration in DMSO. Moxidectin (1% Cydectin Injectable Moxidectin; Boehringer Ingelheim Vetmedica, Ingelheim, Germany) was diluted to a final concentration in DMSO for all feedings. The high-performance liquid chromatography/mass spectroscopy HPLC/MS experiments used either ivermectin (1% Noromectin) or 10 mM ivermectin diluted in DMSO (Selleck Chemicals, Houston, TX). Feeding experiments involved 2 μl of ivermectin or moxidectin diluted in DMSO being added to 998 μl of defibrinated sheep's blood (Hemostat Laboratories, Dixon, CA) before being fed to the insects. Controls were fed 998 μl of sheep's blood plus 2 μl of DMSO. DMSO has previously been shown to cause dose-dependent toxicity in bed bugs and may have contributed to the otherwise higher incapacitation rates in the control groups [8].

High-performance liquid chromatography and mass spectroscopy

Ridge strain *C. lectularius* females and 4th instar nymphs were both fed on blood meals containing 7 ng/mL of ivermectin (1% Normectin) diluted in DMSO and then observed in the laboratory. In the second experiment, males were fed on blood containing 53 ng/mL ivermectin (1% Normectin) or 7 ng/mL ivermectin (Selleckchem) both diluted in DMSO. At specified times after the feedings, live insects were placed at -20 °C until they were ready to be analyzed by HPLC/MS.

Frozen bed bugs were thawed to room temperature, weighed, and 100 μl of 0.2% formic acid was added. Samples were then homogenized. They were then mixed with 1,000 μl of 3:1 volume/volume methanol:acetonitrile, vortexed for 20 seconds, sonicated for 10 minutes, and centrifuged at 16,000 g for 20 minutes. A total of 1,000 μl of supernatant was transferred to a clean tube and speed vacuumed to dryness. It was then reconstituted with 50 μl of reconstitute solvent (0.5 millimolar ammonium formate, 0.1% formic acid in 50% methanol) and then centrifuged at 16,000 g for 20 minutes. Five microliters of the supernatant were used for HPLC and MS. The ivermectin was chromatographically separated from endogenous components on a Waters Atlantis dC18 50 x 2.1 mm, 3 μm column (Waters Corp, Milford, MA) using Agilent 1200 HPLC system (Agilent Technologies, Santa Clara, CA). Mobile phase A was water with 0.5 mM ammonium formate and 0.1% formic acid. Mobile phase B was acetonitrile with 0.1% formic acid. Ivermectin was eluted out of the column by 90% mobile phase B at a flow rate of 0.2 mL/min. For detection and quantitation, we used a Thermo Scientific TSQ Quantum Ultra with HESI-II probe (Thermo Fisher Scientific, Waltham, MA) using electrospray ionization (ESI) positive ionization mode, spray voltage of 3,000 volt, capillary temperature of 200 °C, vaporizer temperature of 300 °C, sheath gas pressure of 40, auxiliary gas pressure of 10, skimmer offset of 10 volt, and standardized mortality ratio set up: Q1: 0.7 full width at half-maximum (FWHM); Q3: 0.7 FWHM; Q2: 1.5 milliTorr (Ar); scan width: 0.002 mass to charge ratio (m/z), and scan time at 0.02 seconds.

Statistical analysis

Continuous variables were summarized with median and range, unless otherwise specified, while categorical variables were summarized with frequency and percent. The Wilcoxon rank-sum test was used to compare the incapacitation rates for *C. lectularius* between bedbugs fed on 0 ng of ivermectin and moxidectin individually with other doses. The Pearson Chi-square test was used to compare female incapacitation rates and first instar proportional differences between moxidectin and ivermectin treatment groups with control. Two multivariable linear regression models for each feeding experiment were created to assess the differences in the percentage of incapacitated bed bugs with respect to their controls (0 ng of ivermectin or moxidectin for each corresponding day) between *C. lectularius* strains. Models were adjusted for the type of drug (ivermectin or moxidectin), the drug dose (e.g., 0, 1, 25, 50), and the number of days after the feeding. Overall group-wise differences were evaluated using the F-test. Estimates of linear change in the percent of incapacitated bed bugs with respect to each strain’s controls for specific variable categories were calculated along with their 95% confidence intervals (CI). All tests were two-sided and p-values less than 0.05 were considered statistically significant. All statistical analysis was performed in R Statistical Software (version 3.4.2; R Foundation for Statistical Computing, Vienna, Austria).

## Results

Effectiveness of ivermectin and moxidectin for incapacitation

Incapacitation rates for the first experiment are summarized below. The mean number of fed insects was 48 [range 10-115; standard deviation (SD): 28]. The median incapacitation rates of all strains on day 11 for bed bugs fed on 25 ng/mL moxidectin (16.3%, range: 8.5-22.2%) were significantly larger than the incapacitation rate of controls (4.1%, range: 0.0-10.2%, p: 0.016). On day 23, the median incapacitation rate for all strains fed on 25 ng/mL ivermectin (36%, range: 23-46%) was significantly larger than the incapacitation rates of the controls (12% range: 0%-22%, p: 0.008). No other differences were observed when compared to controls. (Table 1 and Table 2).

**Table 1:** Incapacitation rates for C. lectularius on moxidectin (first experiment)

	Day 11	Day 11	Day 11	Day 23	Day 23	Day 23
Bed bug strain	0 ng/mL	2.5 ng/mL	25 ng/mL	0 ng/mL	2.5 ng/mL	25 ng/mL
Ridge, %	3.2	5.3	22.2	11.0	5.3	35.2
Anderson, %	0.0	0.0	20.0	0.0	9.5	20.0
Harlan, %	4.1	7.0	16.3	8.2	18.3	23.3
Irvington, %	5.8	4.4	8.5	17.3	13.0	17.0
Indy, %	10.2	9.0	12.3	22.5	20.9	56.2
Median (range), %	4.1 (0.0–10.2)	5.3 (0.0–9.0)	16.3 (8.5–22.2)	11.0 (0.0–22.5)	13.0 (5.3–20.9)	23.3 (17.056.2)
P-value	-	0.75	0.016	-	0.84	0.056

**Table 2:** Incapacitation rates for C. lectularius on ivermectin (first experiment)

	Day 11	Day 11	Day 11	Day 23	Day 23	Day 23
Bed bug strain	0 ng/mL	2.5 ng/mL	25 ng/mL	0 ng/mL	2.5 ng/mL	25 ng/mL
Ridge, %	3.2	NA	34.2	11.0	NA	31.7
Anderson, %	0	3.9	31.8	0.0	15.4	40.9
Harlan, %	4.1	0	0	8.2	2.9	23.1
Irvington, %	5.8	18.8	22.2	17.3	35.4	46.3
Indy, %	10.2	10.9	26.7	22.5	31.5	37.2
Median (range), %	4.0 (0.0–10.2)	7.4 (0.0–18.8)	26.7 (0.0–34.2)	11.0 (0.0–22.5)	23.5 (2.9–35.4)	37.2 (23.1–46.3)
P-value	-	0.54	0.094	-	0.41	0.008

A summary of incapacitation rates for the second experiment can be found below. The mean number of fed bed bugs was 54 (range: 19-102; SD: 19.6). Median incapacitation rates for doses of 25 ng/mL and 50 ng/mL of ivermectin and moxidectin were significantly larger than controls at all measured time points (p: ≤0.012). The minimum incapacitation was 88.0% for moxidectin on day 16, and 52.3% for ivermectin on day 16, compared to the maximum control incapacitation of 45.8% on day 33. Although the incapacitation rates of bed bugs fed on 1 ng/mL of moxidectin was not significantly different on day 3 (41.2%, range: 7.1-63.3%) compared to the controls (8.3%, range: 4.4-25.8%, p: 0.095), with the progression of time, the rates increased to 56.4% (range: 17.9-76.0%) on day 16 and 61.8% (range: 37.5-76.0%) on day 33 (p: ≤0.032). Bed bugs fed on 1 ng/mL ivermectin showed less incapacitation and were comparable to the controls for all time points (p: ≥0.70). (Table 3 and Table 4).

**Table 3:** Incapacitation rates for C. lectularius on moxidectin (second experiment)

	Day 3				Day 16				Day 33				
Bed bug strain	0 ng/mL	1 ng/mL	25 ng/mL	50 ng/mL	0 ng/mL	1 ng/mL	25 ng/mL	50 ng/mL	0 ng/mL	1 ng/mL	25 ng/mL	50 ng/mL
Ridge, %	12.5	7.1	100.0	98.0	29.2	17.9	96.8	88.0	45.8	37.5	96.8	76.0
Anderson, %	25.8	21.1	93.9	97.5	32.3	36.8	100.0	100.0	35.5	47.4	100.0	100.0
Harlan, %	4.4	45.5	96.4	100.0	4.4	56.4	100.0	100.0	15.6	61.8	100.0	100.0
Irvington, %	6.7	63.3	97.1	100.0	8.3	75.9	100.0	100.0	21.7	75.9	98.0	100.0
Indy, %	8.3	41.2	98.9	97.9	11.7	66.2	100.0	100.0	23.3	67.6	100.0	100.0
Median (range), %	8.3 (4.4–25.8)	41.2 (7.1–63.3)	97.1 (93.9–100.0)	98.0 (97.5–100.0)	11.7 (4.4–32.3)	56.4 (17.9–76.0)	100.0 (96.8–100.0)	100.0 (88.0–100.0)	23.3 (15.6–45.8)	61.8 (37.5–76.0)	100.0 (96.8–100.0)	100.0 (76.0–100.0)
P-value	-	0.095	0.008	0.012	-	0.032	0.010	0.010	-	0.016	0.011	0.010

**Table 4:** Incapacitation rates for C. lectularius on ivermectin (second experiment)

	Day 3				Day 16				Day 33				
	0 ng/mL	1 ng/mL	25 ng/mL	50 ng/mL	0 ng/mL	1 ng/mL	25 ng/mL	50 ng/mL	0 ng/mL	1 ng/mL	25 ng/mL	50 ng/mL
Ridge, %	12.5	9.7	68.2	90.5	29.2	27.4	52.3	85.7	45.8	46.8	59.1	73.8
Anderson, %	25.8	3.7	76.5	94.0	32.3	3.7	76.5	98.0	35.5	14.8	52.9	90.0
Harlan, %	4.4	23.3	70.2	87.7	4.4	42.5	77.2	89.5	15.6	39.7	66.7	84.2
Irvington, %	6.7	15.6	90.3	88.9	8.3	28.6	93.5	100.0	21.7	41.6	92.5	96.3
Indy, %	8.3	15.8	89.5	98.3	11.7	21.1	84.2	93.3	23.3	26.3	84.2	86.7
Median (range), %	8.3 (4.4–25.8)	15.6 (3.7–23.3)	76.5 (68.2–90.3)	90.5 (87.7–98.3)	11.7 (4.4–32.3)	27.4 (3.7–42.5)	77.2 (52.3–93.6)	93.3 (85.7–100.0)	23.3 (15.6–45.8)	39.7 (14.8–46.8)	66.7 (52.9–92.5)	86.7 (73.896.3)

Differences in incapacitation rates between *C. lectularius* strains

In the first feeding experiment, Anderson bed bugs were more susceptible and estimated to have experienced 12.47% more incapacitation than Harlan bed bugs (95% CI: 3.47-21.47; p: <0.008). Despite the pairwise difference, group-wise differences between strains remained non-significant (p: 0.062). A dose of 25 ng/mL was associated with a 13.98% higher incapacitation rate than a dose of 2.5 ng/mL (95% CI: 8.08-19.89; p: <0.001) and moxidectin was associated with a 6.18% lower incapacitation rate than ivermectin (95% CI: 0.27-12.09; p: 0.041). (Table 5).

**Table 5:** Regression analysis for differences in incapacitation rates normalized by controls for bed bug strains when controlling for drug, drug dose, and time post-feeding for feeding experiment 1 ref: reference; 95% CI: 95% confidence interval

Variable	Estimated rate of incapacitation, % (95% CI)	P-value
Bed bug Strain (ref: Harlan)	-	-
Anderson	12.47 (3.47–21.47)	0.008
Indy	4.05 (-4.95–13.05)	0.37
Irvington	3.94 (-5.06–12.93)	0.38
Ridge	8.7 (-1.14–18.51)	0.081
Day (ref: Day 11)	-	-
Day 23	4.99 (-0.85–10.83)	0.091
Dose (ref: Dose 2.5)	-	-
25 ng/mL	13.98 (8.08–19.89)	<0.001
Drug (ref: Ivermectin)	-	-
Moxidectin	-6.18 (-12.09 to -0.27)	0.041

In the second feeding experiment, there was an indication of significant group-wise differences between the strains with respect to their controls (p: <0.001) where the Anderson strain was estimated to have an incapacitation rate of 30.49% percentage points less than Harlan (95% CI: 22.43-38.53), and the Ridge strain was estimated to have an incapacitation rate 32.66% points less than Harlan (95% CI: 24.60-40.72). Furthermore, moxidectin showed increased incapacitation rates on average of 17.90% compared to ivermectin (95% CI: 12.81-23.00), and higher doses of both drugs were also associated with increased incapacitation (p: <0.001). (Table 6).

**Table 6:** Regression analysis for differences in incapacitation rates normalized by controls for bed bug strains when controlling for drug, drug dose, and time post-feeding for feeding experiment 2 ref =  reference; 95% CI =  95% confidence interval

Variable	Estimated rate of incapacitation, % (95% CI)	P-value
Bed bug strain (ref: Harlan)	-	-
Anderson	-30.49 (-38.55 to -22.43)	<0.001
Indy	-4.62 (-12.68–3.44)	0.26
Irvington	2.40 (-5.66–10.46)	0.55
Ridge	-32.66 (-40.72 to -24.60)	<0.001
Day (ref: Day 3)	-	-
Day 16	-1.24 (-7.48–5.01)	0.70
Day 33	-12.26 (-18.51 to -6.02)	<0.001
Dose (ref: Dose 1)	-	-
25 ng/mL	50.98 (44.74–57.22)	<0.001
50 ng/mL	57.74 (51.50–63.99)	<0.001
Drug (ref: Ivermectin)	-	-
Moxidectin	17.90 (12.8123.00)	<0.001

The fecundity data for experiment two with all bed bug strains combined with drug and drug concentration are given below. There were no eggs or new 1st instars at day 33 in either the 25 ng/mL or 50 ng/mL ivermectin or moxidectin 50 ng/mL groups compared with 97 eggs and 55 new 1st instars in the control group. On day 33, the percentage of new 1st instars laid by adult females was 57%, 38%, and 73% for the control group, 1 ng/mL moxidectin, and 1 ng/mL ivermectin, respectively. The number of new 1st instars per alive adult females at day 33 was 2.5, 1.4, and 3.1 for the control group, 1 ng/mL moxidectin, and 1 ng/mL ivermectin, respectively. There were no eggs laid or new 1st instars for the moxidectin and ivermectin at 25 ng/mL and 50 ng/mL groups. (Table 7).

**Table 7:** Combined incapacitation rates and fecundity for all C. lectularius strains on day 33 on different quantities of moxidectin or ivermectin n: population size affected; N: total population size; NA: not applicable *P-values measure the significance in the differences between moxidectin and ivermectin with control using the Pearson Chi-square test

Drug	Dose	First instars per number of live adult females	Adult female incapacitation		First instars per number of laid eggs	
	(ng/mL)	Ratio	% (n/N)	P-value*	% (n/N)	P-value*
Control	0	2.5	24 (7/29)	NA	57 (55/97)	NA
Moxidectin	1	1.4	50 (19/38)	0.031	38 (26/68)	0.020
Moxidectin	25	0	98 (53/54)	<0.001	0 (0/1)	0.26
Moxidectin	50	0	88 (44/50)	<0.001	NA	NA
Ivermectin	1	3.1	18 (9/50)	0.51	73 (125/171)	0.006
Ivermectin	25	0	77 (24/31)	<0.001	NA	NA
Ivermectin	50	0	93 (43/46)	<0.001	NA	NA

High-performance liquid chromatography/mass spectroscopy

The concentration of ivermectin after one day of feeding on 7 ng/mL ivermectin as measured by HPLC/MS averaged 3.64 ng/mL and 2.13 ng/mL for two 4th instar nymphs and two females, respectively. The mean ivermectin concentration in two adult females *C. lectularius* was 0.65 ng/mL 14 days after feeding on a blood meal spiked with 7 ng/mL of ivermectin. The mean ivermectin concentration in two 4th stage instar nymphs *C. lectularius* was 0.58 ng/mL for two 4th stage instar nymphs and 0.55 ng/mL for two females 17 days after feeding on a blood meal spiked with 7 ng/mL of ivermectin. The mean ivermectin concentration in one 4th stage instar nymphs *C. lectularius* was 0.62 ng/mL 30 days after feeding on a blood meal spiked with 7 ng/mL of ivermectin.

For feeding experiment 2, the mean concentration of ivermectin in three males was 37.67 ng/mL and 5.03 ng/mL immediately after feeding on a blood meal containing 53 ng/mL or 7 ng/mL ivermectin, respectively. A single male subject had an ivermectin concentration of 0.48 ng/mL seven days after feeding on a blood meal containing 7 ng/mL ivermectin.

The concentration of ivermectin in bed bugs goes down more quickly during the first week but was relatively stable between 1-4 weeks post-feeding. The drug was still detectable in the insect one month after the feeding. The long-term persistence of ivermectin in the bed bug may help explain why the insects that survive a sublethal dose of ivermectin can manifest long-term morbidity.

## Discussion

Pyrethroid sensitivity has not been determined for either the Anderson or Ridge strains of *C. lectularius*, but we found no difference in incapacitation rates between the pyrethroid-sensitive Harlan and the reported pyrethroid-resistant Indy and Irvington strains in either feeding experiment. In our second feeding experiment, all groups suffered higher incapacitation rates when fed on blood containing higher concentrations of either moxidectin or ivermectin. Also in experiment 2, all strains that fed on ≥25 ng/mL of ivermectin and moxidectin suffered significant harm including the prevention of egg-laying and the production of eclosed 1st instars. The harm caused by ivermectin and moxidectin persisted until at least day 33.

The pyrethroid-class of pesticides has a different mode of action than the macrocyclic lactones, which include ivermectin and moxidectin. Pyrethroids are axonic excitotoxins that prevent the closure of the voltage-gated sodium channels in insect axonal membranes. Ivermectin and moxidectin act on the invertebrate glutamate-gated chloride channels [and especially for moxidectin in the gamma-aminobutyric (GABA) mediated chloride channels] in nerve and muscle cells leading to increased chloride permeability and cellular hyperpolarization and paralysis [12].

This study had several limitations; ivermectin and moxidectin were diluted in DMSO and added to blood samples and fed to the insects in the laboratory in our study. When administered *in vivo,* ivermectin has metabolites that are toxic to bed bugs, which could not be detected in our experiments [6,10]. Mortality in our control groups was higher than what would be expected from feeding bed bugs and may represent some DMSO toxicity [8]. Pyrethroid-resistance levels in the insects were not confirmed prior to their use in the experiments. Laboratory strains of bed bugs were used and pyrethroid-resistance could have waned over time without repeated exposure to pyrethroids. Lastly, all the life stages of the bed bugs were not recorded; so we were unable to ascertain from these experiments if ivermectin and moxidectin adversely affected all life stages similarly; however, this has been examined previously [5,7,9-10].

A randomized double-blind placebo-controlled clinical trial that involves giving ivermectin or moxidectin to humans, along with IPM in both arms, would be helpful in showing whether these drugs could be helpful in eliminating a bed bug infestation. A single dose of ivermectin or moxidectin administered without IPM for bed bug control is not likely to be effective, and oral anti-parasitic drugs would not likely be indicated for many home bed bugs infestations. However, some bed bug infestations are difficult to control using standard IPM techniques and the addition of oral anti-parasitics under these circumstances might aid in the elimination of insect infestation. *C. lectularius* feeds about every 2.5 days and eggs take about a week to hatch [14]. The pharmacokinetics of ivermectin and moxidectin are such that a single dose of either drug would not likely control an established infestation when used in isolation. However, a single 0.2-mg/kg dose of ivermectin in humans has been shown to cause long-term harm to bed bug populations that fed on the person’s blood shortly after consuming the drug [10]. Higher and more frequent doses of ivermectin have been shown to be safe in humans, and ivermectin is currently being evaluated for control of mosquito-borne diseases [15-20].

We conducted the experiments using HPLC/MS to measure ivermectin inside *C. lectularius*. Our preliminary results suggested that the concentration of ivermectin in bed bugs went down more quickly during the first week but was relatively stable between 1 and 4 weeks post-feeding. The drug was still detectable in the insect one month after the feeding. The long-term persistence of ivermectin in the bed bug may help explain why the insects that survive a sublethal dose of ivermectin can manifest long-term morbidity [6,7,9-10]. The ivermectin fed to bed bugs in our experiments was diluted in DMSO, an aprotic solvent miscible in many organic solvents and water; hence, future experiments should ideally measure ivermectin levels in bed bugs for longer periods of time, from multiple *C. lectularius* strains, and from *in vivo* ivermectin blood meals.

## Conclusions

We are able to conclude that Ivermectin and moxidectin cause harm to multiple strains of *C. lectularius*. Laboratory feeding experiments involving ivermectin or moxidectin failed to demonstrate significant differences in the incapacitation rates between the pyrethroid-sensitive Harlan and pyrethroid-resistant Indy and Irvington *C. lectularius* strains. The persistence of ivermectin inside fed bed bugs could potentially help explain the long-term harm to the insect after feeding on blood meals containing ivermectin.
